# Three-Body Excitations in Fock-Space Coupled-Cluster: Fourth Order Perturbation Correction to Electron Affinity and Its Relation to Bondonic Formalism

**DOI:** 10.3390/ijms22168953

**Published:** 2021-08-19

**Authors:** Suhita Basumallick, Mihai V. Putz, Sourav Pal

**Affiliations:** 1 Department of Chemistry, Indian Institute of Technology Bombay, Powai, Mumbai 400076, India; basumallick.suhita16@gmail.com; 2 Laboratory of Structural and Computational Physical-Chemistry for Nanosciences and QSAR, Biology-Chemistry Department, Faculty of Chemistry, Biology, Geography, West University of Timisoara, Str. Pestalozzi No. 16, 300115 Timisoara, Romania; 3 Department of Chemical Sciences, Indian Institute of Science Education and Research Kolkata, Mohanpur, Nadia 741246, India

**Keywords:** electronic affinity, Fock-space coupled-cluster theory, perturbative triples, multi-reference coupled-cluster, bondonic formalism

## Abstract

In this paper, we present a formulation of highly correlated Fock-space multi-reference coupled-cluster (FSMRCC) methods, including approximate triples on top of the FSMRCC with singles and doubles, which correct the electron affinities by at least at third and up to the fourth order in perturbation. We discuss various partial fourth-order schemes, which are reliable and yet computationally more efficient than the full fourth-order triples scheme. The third-order scheme is called MRCCSD+T^*^(3). We present two approximate fourth-order schemes, MRCCSD+T^*^−a(4) and MRCCSD+T^*^(4). The results that are presented allow one to choose an appropriate fourth-order scheme, which is less expensive and right for the problem. All these schemes are based on the effective Hamiltonian scheme, and provide a direct calculation of the vertical electron affinities. We apply these schemes to a prototype Li_2_ molecule, using four different basis sets, as well as BeO and CH^+^. We have calculated the vertical electron affinities of Li_2_ at the geometry of the neutral Li_2_ molecule. We also present the vertical ionization potentials of the Li_2_ anion at the geometry of the anion ground state. We have also shown how to calculate adiabatic electron affinity, though in that case we lose the advantages of direct calculation. BeO has been examined in two basis sets. For CH^+^, four different basis sets have been used. We have presented the partial fourth-order schemes to the EA in all the basis sets. The results are analyzed to illustrate the importance of triples, as well as highlight computationally efficient partial fourth-order schemes. The choice of the basis set on the electron affinity calculation is also emphasized. Comparisons with available experimental and theoretical results are presented. The general fourth-order schemes, which are conceptually equivalent with the Fock-space multi-reference coupled-cluster singles, doubles, and triplets (MRCCSD+T) methods, based on bondonic formalism, are also presented here in a composed way, for quantum electronic affinity.

## 1. Introduction

Many electron systems often require improved electron correlation, for a quality description of the wave function, in order to understand the structure and properties of these systems. Moreover, the numerical cost of electron correlation calculation grows rapidly with the size of the basis set. Therefore, effective approximate methods have to be proposed, as a compromise between the computational cost of evaluating electron correlation and the quality that is required of the wave function [1,2,3,4,5,6,7].

Quantitative investigations, for understanding phenomena such as the electron-donating properties of ligands in coordination compounds, the energy changes occurring in oxidation–reduction reactions and in charge transfer reactions, the calculation of lattice energies from the Born–Haber cycle, and the inductive effects of different groups in chemical bonding, require a knowledge of the electron affinity [8,9,10,11,12].

There is a large number of experimental techniques that have been applied to the determination of electron affinities, such as laser photoelectron spectroscopy and electron transmission spectroscopy [13,14].

The computational methods require flexible basis sets and careful treatment of electron correlation, for a systematic evaluation of the electron affinities (EA) [15,16]. 

It is a challenging task, even for ab initio methods, because the energy of interest is a very small fraction of the total electronic energy of the parent neutral system. Thus, EA is a sensitive quantity, which requires the inclusion of higher-order electron correlation methods, along with basis sets that are large enough, and can be a good testing ground for the accuracy of approximate theoretical models. At the same time, the quantum theory, by merging the fermionic and bosonic features of matter, offers a description of the chemical bond where bondons act as virtual quantum particle. Bondonic chemistry promotes the modeling of chemical transformations by the quantum particle of the chemical field, called bondons, rather than by the molecular wave function.

The single-reference coupled-cluster (SRCC) method was introduced in the early 1960s, and over the last two decades it has become the most powerful approach in the field, for its capability of predicting the properties of systems to a high degree of accuracy [2,3,17,18,19,20,21,22,23,24,25,26]. It treats the dynamical electron correlation problem, in terms of an infinite-order exponential ansatz of interacting clusters of electrons. Further, the CCSD(T) method [27,28,29,30,31,32], which is an extension of the standard coupled-cluster singles and doubles model, has proved to be the current “gold standard” of ab initio quantum chemistry, due to its success in chemical applications. Though, spin orbital-based coupled-cluster methods allow the use of a wide range of reference functions, for open-shell system coupled-cluster wave functions based on ROHF, or when the UHF reference function fails to be a rigorous eigen function of the S^2^ operator. Thus, Fock-space coupled-cluster (FSCC) [33,34,35,36,37,38,39,40,41,42,43,44], on the other hand, is an approach to an open-shell system, which is capable of providing a true eigen function of the S^2^ operator, and has the ability to handle the multi-reference determinants that are needed to represent quasi-degenerate states. Moreover, the FSCC method is capable of providing energy differences for several states, in a single calculation, at a lesser computational cost, and offers a distinct advantage over the SRCC approach. The Fock-space-based approach, to correlate model spaces that have different numbers of electrons, also has the advantage of the calculation of EA in a direct manner. Comprehensive discussions on FSCC are presented in the references [40,45,46]. The FSCC, in its singles and doubles model (FSCCSD), has been well developed and studied for direct difference energies [40,41,42], as well as for energy derivatives, by Pal and co-workers [47,48,49]. They have formulated a linear response approach [47], followed by the evolution of a Z-vector-type approach [48], with the idea of Lagrange multipliers [49], within FSMRCC method, in order to get a satisfactory result for the calculation of energy derivatives, including properties such as the dipole moments and polarizabilities of molecules. Their approach is not only applicable for complete model spaces, but it is also applicable for incomplete model spaces. Moreover, the implementations of the complex absorbing potential (CAP) in the FSMRCC framework for resonant states, have also been achieved by Pal and co-workers [46,50,51,52].

Although FSCC is a post-CC method, as the FSCC equations depend on the solved CC energy and wave function, for a given truncation, both FSCC and CC do not provide equivalent quality results, due to the restriction of the excitations from the inactive to active orbital space in the FSCC method. Therefore, for a given truncation, to match the expansion space, the FSCC calculation must include spectator contributions from higher cluster operators. Thus, to improve the quality of FSCCSD, we have to include triple excitation effects (EE) in FSCCSD (FSCCDT), to gain accurate theoretical results. The requirement of non-spectator triples is often very important and it significantly changes the results. However, the full FSCCSDT model [53,54] is computationally expensive. Therefore, as a compromise, several approximations to include triples are required [37,55,56,57]. Pal et al., in their earlier work, have included triples excitations at various approximate levels for IP and EE [55,57].

We may mention that the equation-of-motion coupled-cluster (EOM-CC) [58,59] is an alternative development to the FSCC method, in that it yields the difference energies directly. There are versions of EOM-CC [60] for the calculation of IP, EA [61], and EE [62]. Specifically, the EOM-CC for EA is known as EA-EOM-CC; one may add that the IP- and EA-EOM-CC energies are equivalent to the one-hole and one-particle FS sector energies, respectively. Substantial developments of EOM-CC have taken place in singles and doubles, as well as with full triples and some perturbative triples [63]. In the context of the EOM-CC, it obviously improves the results. Fundamentally, EOM-CC is different from FS-CC, in the sense that only a single determinant is used for the ground state, and the target state is described by the action of a linear operator. The analytic gradients of EOM-CC have also been developed [64]. While the Fock space suffers from intruder states in some critical cases, strategies of intermediate Hamiltonian have been formulated to overcome this [65]. EOM-CC, on the other hand, does not suffer from this problem. However, it is well known that EOM-CC does not provide correct scaling of difference energies. One requires another similarity transformation, in what is known as ST-EOM-CC, to achieve this. This is more closely related to FS-CC [66]. Within the EOM-CC context, full methods [67], as well as one with selected triples [68] and an approximate EOM-CCSDT-3 [69], have been developed.

In the present work, we report the development of a computer code of perturbative approximation to a FSCCSDT model, using a primarily non-iterative or single-iteration approach for the inclusion of triples up to third and fourth order. Similar efforts were made for low-order improvements to ionization potentials, but not for electron affinities, following the work of Pal et al. [56]. Within the intermediate Hamiltonian version, the work of Musial and co-workers, in the inclusion of triples, should also be mentioned [70]. However, compared to all these developments, the novel aspect of the work that is presented here is the partial fourth-order schemes, which have only been attempted recently by us [71], in the context of IP calculations; with that occasion, the bondonic formalism was also illustrated for the first time, for treating such higher-order perturbation excitation effects.

Such efforts, in the context of EOM-CC, have not been carried out and, in fact, no parallel to this, in the context of EOM-CC, can be made. In the context of EA calculations, which are computationally more demanding, such efforts are required even more. We have reported precisely such studies of systematic improvements to EA, by adding triples corrections to at least third-order schemes, and then through two partial and full fourth-order schemes, due to the triples. The full scheme needs only one iteration of selected diagrams that fully correct to at least up to the fourth order and other third order, and the two partial fourth-order schemes are completely non-iterative [55,57]. These two non-iterative partial fourth-order schemes are computationally less expensive, and yet provide results that are closer to the full triples-corrected fourth-order scheme. The two partial fourth-order schemes, called MRSSCD+T^*^−a(4) and MRCCS+T^*^−b(4), are the important approximations presented in this manuscript. Among these, the first one is even more computationally efficient. We highlight, from the numerical results, that the first partial scheme can often be enough to get significantly improved results. One can thus choose triples-corrected fourth-order schemes, depending on the size of the problem. Different strategies may be implemented, in terms of computer time and storage, to include such effects.

We have also reported concepts related to the algorithm that has been adopted, in order to optimize the computational requirements. We may mention that the scheme to calculate the EA of a molecule can be used to generate results for the ionization potential of the corresponding molecular anion. Since the calculations are direct, these are amenable to vertical difference energy calculations. We have also shown how to calculate adiabatic EA, although this will no longer be a direct calculation (it could be direct at each geometry).

In Section 2, we summarize the FSCC theory for the (1,0) sector. This will help us to introduce the background for the present work, and we also present the equations for the inclusion of the triply excited amplitudes, to generate EAs that are correct up to the third, as well as fourth, order. In Section 3, we report the details of the triples correction at the third and fourth order. In Section 5, we describe the implementation of these to the computer code. In Section 4, the analogous fourth-order schemes, which are conceptually equivalent with MRCCSD+T methods, based on bondonic formalism, are reported for electron affinity. In Section 6, we present some model results of the two molecules Li_2_ and BeO, in different basis sets. We present the vertical EA of Li_2_, vertical IP of the Li_2_ anion (at the anion geometry), as well as the adiabatic EA of Li_2_, in four different bases. In the same section, we present the results for BeO in two different basis sets, as well as the vertical EA of CH^+^. We compare these with the earlier results, using singles and doubles amplitudes only, and experimental values. Section 7 presents a perspective on computational time, with conclusions in Section 8.

## 2. Theory Description: Basis Structure

The traditional Fock-space approach is based upon a valence universal wave operator Ω, which, when acting on an appropriate model space, yields the desired wave function. The theory is based on a common vacuum, which defines holes and particles. Usually, an appropriate closed-shell single-determinant restricted Hartree–Fock (RHF) wave function is chosen as a vacuum. An appropriate Fock space, consisting of m holes and n particles (m,n sector), is defined for a specific problem. This also subdivides the holes and particles into active and inactive subparts. For example, for electron-attached states, a suitable Fock space consists of one particle, with respect to the RHF of the neutral ground state as a vacuum. This will thus define the (1,0) Fock space sector. The FSCC model space is defined as follows:

(1) $$\left|\psi_{i}^{0\left(m,n\right)}\rangle \right.=\underset{\mu }{\sum }c_{\mu i}\left|\varphi_{\mu }^{\left(m,n\right)}\rangle \right.$$

(2) $$\left|\psi_{i}^{\left(m,n\right)}\rangle \right.=\Omega \left|\psi_{i}^{0\left(m,n\right)}\rangle \right.$$

With $\Omega =\left\{e^{\hat{T}}\right\}$, and where $\varphi_{\mu }^{\left(m,n\right)}$ defines the determinants within active particles and active holes. For the (m,n) sector, ${\hat{T}}^{\left(m,n\right)}=\sum_{\begin{array}{c} k=0\ldots m\\ l=0\ldots n \end{array}}{\hat{T}}^{\left(k,l\right)}.\;{\hat{T}}^{\left(k,l\right)}$ annihilates specifically k active particles and l active holes, in addition to other hole-particle creations. Thus, the (m,n) sector correlation operator consists of operators of all the lower sectors. The Bloch equation is used to solve the set of equations. Normal ordering of the wave operator, as proposed by Lindgren [72], ensures decoupling of the equations, such that a specific Bloch equation contains operators of that sector and lower. For the specific (1,0) sector, the ${\hat{T}}^{\left(1,0\right)}$ equation depends on the ${\hat{T}}^{\left(0,0\right)}$ and ${\hat{T}}^{\left(1,0\right)}$ operators. The second-quantized form of operators in singles and doubles may be represented as follows.

For the (0,0) sector, the following:

(3a) $${\hat{T}}_{1}^{\left(0,0\right)}=\underset{i,a}{\sum }t_{i}^{a}\left\{{\hat{a}}^{†}\hat{i}\right\}$$

(3b) $${\hat{T}}_{2}^{\left(0,0\right)}=\frac{1}{4}\underset{\begin{array}{c} i,a\\ j,b \end{array}}{\sum }t_{\mathrm{ij}}^{\mathrm{ab}}\left\{{\hat{a}}^{†}{\hat{b}}^{†}\hat{j}\hat{i}\right\}$$

For the (1,0) sector, the following:

(3c) $${\hat{T}}_{1}^{\left(1,0\right)}=\sum_{a}^{\mathrm{active}}\sum_{b}^{\mathrm{inactive}}t_{a}^{b}\left\{{\hat{b}}^{†}\hat{a}\right\}$$

(3d) $${\hat{T}}_{2}^{\left(1,0\right)}=\frac{1}{2}\overset{\mathrm{active}}{\underset{a}{\sum }}\underset{b}{\sum }\underset{k}{\sum }\underset{c}{\sum }t_{\mathrm{ak}}^{\mathrm{bc}}\left\{{\hat{b}}^{†}{\hat{c}}^{†}\hat{k}\hat{a}\right\}$$

Similarly, three body operators may be defined. The Bloch equation is defined as follows:

(4a) $$P^{\left(k,l\right)}\left[H\Omega -{\Omega H}_{\mathrm{eff}}\right]P^{\left(k,l\right)}=0$$

(4b) $$Q^{\left(k,l\right)}\left[H\Omega -{\Omega H}_{\mathrm{eff}}\right]P^{\left(k,l\right)}=0$$

where k = 0, 1, 2, …, m and l = 0, 1, 2, …, n. The above structure, with the normal ordering of the wave operator, suggests that the equations can be solved from the lowest sector upwards. Since the (1,0) Fock space is a complete Fock space by definition, PΩP = P, and thus the first Equation (4a) is the defining equation for the effective Hamiltonian within the FS sector (P$H_{eff}$P). Equation (4b) solves the amplitudes of Ω, using the subsystem embedding condition (SEC) [73]. Specifically, for the (1,0) sector, the (0,0) sector, which is the standard single-reference CC theory, is first solved, before the ${\hat{T}}^{\left(1,0\right)}$ amplitudes are obtained. For computational simplicity, ${\left(H\Omega \right)}_{c}\equiv {\left({\mathrm{He}}^{T^{\left(0,0\right)}}e^{T^{\left(1,0\right)}}\right)}_{c}$ is split into $\bar{H}={\left({\mathrm{He}}^{T^{\left(0,0\right)}}\right)}_{c}$ and ${\left(H\Omega \right)}_{c}\equiv {\left({\bar{H}e}^{T^{\left(1,0\right)}}\right)}_{c}$. $\bar{H}$ is the effective operator, which is constructed after the $e^{T^{\left(0,0\right)}}$ amplitudes are obtained. $\bar{H}$ has many different body parts and can be written as follows:

(4c) $$\bar{H}={\bar{f}}_{N}+{\bar{v}}_{N}+{\bar{w}}_{N}+\cdots$$

where ${\bar{f}}_{N},{\bar{v}}_{N},\;\mathrm{and}\;{\bar{w}}_{N}$ are one, two, and three body parts, respectively. The Bloch equation for the (1,0) sector can be rewritten in the CCSD approximation as follows:

(5) $${\hat{H}}_{\mathrm{eff}}^{\left(1,0\right)}={\hat{P}}^{\left(1,0\right)}\left\{{\bar{f}}_{N}\left(1+{\hat{T}}_{1}^{\left(1,0\right)}+{\hat{T}}_{2}^{\left(1,0\right)}\right)+{\bar{v}}_{N}{\hat{T}}_{2}^{\left(1,0\right)}\right\}{\hat{P}}^{\left(1,0\right)}$$

(6) $${\hat{Q}}_{1}^{\left(1,0\right)}\left\{{\bar{f}}_{N}\left(1+{\hat{T}}_{1}^{\left(1,0\right)}+{\hat{T}}_{2}^{\left(1,0\right)}\right)+{\bar{v}}_{N}{\hat{T}}_{2}^{\left(1,0\right)}-{\hat{T}}_{1}^{\left(1,0\right)}{\hat{H}}_{\mathrm{eff}}^{\left(1,0\right)}\right\}{\hat{P}}^{\left(1,0\right)}=0$$

(7) $${\hat{Q}}_{2}^{\left(1,0\right)}\left\{{\bar{f}}_{N}{\hat{T}}_{2}^{\left(1,0\right)}+{\bar{v}}_{N}\left(1+{\hat{T}}_{1}^{\left(1,0\right)}+{\hat{T}}_{2}^{\left(1,0\right)}\right)+{\bar{w}}_{N}{\hat{T}}_{2}^{\left(1,0\right)}-{\hat{T}}_{2}^{\left(1,0\right)}{\hat{H}}_{\mathrm{eff}}^{\left(1,0\right)}\right\}{\hat{P}}^{\left(1,0\right)}=0$$

Initially, a ground-state CCSD calculation has to be performed to obtain the converged amplitudes ${\hat{T}}_{1}^{\left(0,0\right)}$ and ${\hat{T}}_{2}^{\left(0,0\right)}$. From these converged amplitudes and the molecular orbital integrals, the elements of the effective CC Hamiltonian $\bar{H}$ can be obtained.

As was discussed, the FSCC method and EOM-CC method have similarities. The FS method plugs an effective Hamiltonian of small dimension, unlike the EOM-CC, which is the origin of intruder states; this leads to problems in the convergence of the non-linear equations. Methods, based on intermediate Hamiltonian, have been formulated to overcome this [65].

## 3. Approximate Triplets: Perturbative Analysis

In this section, we discuss the approximate inclusion of triples in our FS-MRCCSD implementation of EA. The full FSCCSDT equations can then be written as follows:

(8) $${\hat{H}}_{\mathrm{eff}}^{\left(1,0\right)}={\hat{P}}^{\left(1,0\right)}\left\{{\bar{f}}_{N}\left(1+{\hat{T}}_{1}^{\left(1,0\right)}+{\hat{T}}_{2}^{\left(1,0\right)}\right)+{\bar{v}}_{N}\left({\hat{T}}_{2}^{\left(1,0\right)}+{\hat{T}}_{3}^{\left(1,0\right)}\right)+{\bar{w}}_{N}{\hat{T}}_{3}^{\left(1,0\right)}\right\}{\hat{P}}^{\left(1,0\right)}$$

(9) $${\hat{Q}}_{1}^{\left(1,0\right)}\left\{{\bar{f}}_{N}\left(1+{\hat{T}}_{1}^{\left(1,0\right)}+{\hat{T}}_{2}^{\left(1,0\right)}\right)+{\bar{v}}_{N}\left({\hat{T}}_{2}^{\left(1,0\right)}+{\hat{T}}_{3}^{\left(1,0\right)}\right)+{\bar{w}}_{N}{\hat{T}}_{3}^{\left(1,0\right)}-{\hat{T}}_{1}^{\left(1,0\right)}{\hat{H}}_{\mathrm{eff}}^{\left(1,0\right)}\right\}{\hat{P}}^{\left(1,0\right)}=0$$

(10) $${\hat{Q}}_{2}^{\left(1,0\right)}\left\{{\bar{f}}_{N}\left({\hat{T}}_{2}^{\left(1,0\right)}+{\hat{T}}_{3}^{\left(1,0\right)}\right)+{\bar{v}}_{N}\left(1+{\hat{T}}_{1}^{\left(1,0\right)}+{\hat{T}}_{2}^{\left(1,0\right)}+{\hat{T}}_{3}^{\left(1,0\right)}\right)+{\bar{w}}_{N}\left({\hat{T}}_{2}^{\left(1,0\right)}+{\hat{T}}_{3}^{\left(1,0\right)}\right)-{\hat{T}}_{2}^{\left(1,0\right)}{\hat{H}}_{\mathrm{eff}}^{\left(1,0\right)}\right\}{\hat{P}}^{\left(1,0\right)}=0$$

(11) $${\hat{Q}}_{3}^{\left(1,0\right)}\left\{{\bar{f}}_{N}{\hat{T}}_{3}^{\left(1,0\right)}+{\bar{v}}_{N}\left({\hat{T}}_{2}^{\left(1,0\right)}+{\hat{T}}_{3}^{\left(1,0\right)}\right)+{\bar{w}}_{N}\left(1+{\hat{T}}_{1}^{\left(1,0\right)}+{\hat{T}}_{2}^{\left(1,0\right)}+{\hat{T}}_{3}^{\left(1,0\right)}\right)-{\hat{T}}_{3}^{\left(1,0\right)}{\hat{H}}_{\mathrm{eff}}^{\left(1,0\right)}\right\}{\hat{P}}^{\left(1,0\right)}=0$$

The above expressions suggest that the full inclusion of triples is very expensive, and thus we propose approximate triples for both the (0,0) and (1,0) sectors. The approximation is motivated by a perturbative analysis [47]. A similar analysis exists for the ionization potential [52]. Furthermore, we start from the SRCC similarity-transformed Hamiltonian, and this (0,0) sector has been kept truncated at the singles and doubles labels. Then, we solve the FSCC equations for the state of interest of a given Fock-space sector. The original problem is decoupled into sup problems, due to normal ordering. In order to consider a balanced correlation for the entire wave function, we impose inclusion of the ${\hat{T}}_{3}^{\left(0,0\right)}$ into ${\hat{T}}_{2}^{\left(0,0\right)}$, triples at the (0,0) sector as well as the (1,0) sector, correcting effective Hamiltonian at both the third and fourth orders. This inclusion of triples is on and above the full MRCCSD, which implies the effects of one and two body operators, included up to infinite order in the perturbation. Further, we will see that the triples affect the singles and doubles amplitudes, to the extent of providing corrections to the effective Hamiltonian at the fourth order. Such effects have normally been included. To analyze the effect of perturbative approximate triples to ${\hat{H}}_{\mathrm{eff}}^{\left(1,0\right)}$, let us consider the expression of ${\hat{H}}_{\mathrm{eff}}^{\left(1,0\right)}$, ${\hat{T}}_{3}^{\left(1,0\right)}$, ${\hat{T}}_{1}^{\left(1,0\right)}$, ${\hat{T}}_{2}^{\left(1,0\right)}$, up to lowest order forms, as follows:

(12) $${\hat{H}}_{\mathrm{eff}}^{\left(1,0\right)}={\hat{P}}^{\left(1,0\right)}\left\{{\bar{f}}_{N}+{\bar{f}}_{N}{\hat{T}}_{1}^{\left(1,0\right)}+{\bar{f}}_{N}{\hat{T}}_{2}^{\left(1,0\right)}+{\bar{v}}_{N}{\hat{T}}_{2}^{\left(1,0\right)}+{\bar{v}}_{N}{\hat{T}}_{3}^{\left(1,0\right)}\right\}{\hat{P}}^{\left(1,0\right)}$$

(13) $${\hat{Q}}_{3}^{\left(1,0\right)}\left\{{\bar{f}}_{N}{\hat{T}}_{3}^{\left(1,0\right)}+{\bar{v}}_{N}{\hat{T}}_{2}^{\left(1,0\right)}+{\bar{w}}_{N}-{\hat{T}}_{3}^{\left(1,0\right)}{\bar{H}}^{\left(1,0\right)}\right\}{\hat{P}}^{\left(1,0\right)}=0$$

(14) $${\hat{Q}}_{1}^{\left(1,0\right)}\left\{{\bar{f}}_{N}+{\bar{f}}_{N}{\hat{T}}_{1}^{\left(1,0\right)}+{\bar{f}}_{N}{\hat{T}}_{2}^{\left(1,0\right)}+{\bar{v}}_{N}{\hat{T}}_{2}^{\left(1,0\right)}+{\bar{v}}_{N}{\hat{T}}_{3}^{\left(1,0\right)}-{\hat{T}}_{1}^{\left(1,0\right)}{\hat{H}}_{\mathrm{eff}}^{\left(1,0\right)}\right\}{\hat{P}}^{\left(1,0\right)}=0$$

(15) $${\hat{Q}}_{2}^{\left(1,0\right)}\left\{{\bar{f}}_{N}{\hat{T}}_{2}^{\left(1,0\right)}+{\bar{v}}_{N}+{\bar{v}}_{N}{\hat{T}}_{2}^{\left(1,0\right)}+{\bar{v}}_{N}{\hat{T}}_{3}^{\left(1,0\right)}+{\bar{w}}_{N}{\hat{T}}_{2}^{\left(1,0\right)}-{\hat{T}}_{2}^{\left(1,0\right)}{\hat{H}}_{\mathrm{eff}}^{\left(1,0\right)}\right\}{\hat{P}}^{\left(1,0\right)}=0$$

From Equation (12), we observe that the third order correction, due to triples, is explicitly due to the ${\hat{P}}^{\left(1,0\right)}{\bar{v}}_{N}{\hat{T}}_{3}^{\left(1,0\right)}{\hat{P}}^{\left(1,0\right)}$ term. For this, however, it suffices to take ${\hat{T}}_{3}^{\left(1,0\right)}$ up to the second order in Equation (13).

(16) $${\hat{Q}}_{3}^{\left(1,0\right)}\left\{{\bar{f}}_{N}{\hat{T}}_{3}^{\left(1,0\right)}+{\bar{v}}_{N}{\hat{T}}_{2}^{\left(1,0\right)}+{\bar{w}}_{N}-{\hat{T}}_{3}^{\left(1,0\right)}{\bar{f}}_{N}\right\}{\hat{P}}^{\left(1,0\right)}=0$$

where ${\hat{H}}_{\mathrm{eff}}^{\left(1,0\right)}$ has been replaced by ${\bar{f}}_{N}$. Further, a diagonal approximation of ${\bar{f}}_{N}$ suffices. Hence, using this, we can calculate ${\hat{T}}_{3}^{\left(1,0\right)}$ non-iteratively, by taking the values of ${\hat{T}}_{2}^{\left(1,0\right)}$ that were obtained from the MRCCSD equation of the (1,0) sector, and ${\bar{w}}_{N}$ up to the second order. This also implies that while ${\hat{T}}_{3}^{\left(1,0\right)}$ is correct up to the second order, partial higher order corrections are also taken into account, due to the higher-order effects in ${\hat{T}}_{2}^{\left(1,0\right)}$ and ${\hat{T}}_{1}^{\left(1,0\right)}$ from the MRCCSD equation. The construction of ${\bar{w}}_{N}$ up to the second order is conducted when required. This scheme is called the MRCCSD+$T^{*}\left(3\right)$ scheme.

We now explain the various fourth order schemes, due to the triples that we have proposed. First, we include that the effects of ${\hat{T}}_{3}^{\left(1,0\right)}$, calculated up to the second order in Equation (15), affect the ${\hat{T}}_{2}^{\left(1,0\right)}$ amplitudes at the third order. Clearly, this will affect the effective Hamiltonian at the fourth order, and this scheme has been called MRCCSD+T^*^−a(4), which is a partial fourth-order correction, due to the triples. Subsequently, we go back to Equation (13) and include ${\bar{w}}_{N}$ to at least the third order, by taking the $v_{N}{\hat{T}}_{2}^{\left(0,0\right)}{\hat{T}}_{2}^{\left(0,0\right)}$ term, in addition to the $v_{N}{\hat{T}}_{2}^{\left(0,0\right)}$ term. This now includes the ${\bar{w}}_{N}$, fully corrected up to at least the third order. Using this, as well as ${\bar{w}}_{N}{\hat{T}}_{2}^{\left(1,0\right)}$, with the new values of ${\hat{T}}_{2}^{\left(1,0\right)}$, as in the MRCCSD+T^*^−a(4) scheme, we calculate ${\hat{T}}_{3}^{\left(1,0\right)}$. This partially corrects ${\hat{T}}_{3}^{\left(1,0\right)}$ up to the third order. It is important to emphasize that, at this stage, the corrections in the triples are still essentially non-iterative. The effective Hamiltonian generated at this level, is only partially correct up to the fourth order, since Equation (13) has the a ${\bar{v}}_{N}{\hat{T}}_{3}^{\left(1,0\right)}$ term, which has been omitted at this stage. This is called MRCCSD+T^*^−b(4). Finally, in Equation (13), the term of $v_{N}{\hat{T}}_{3}^{\left(0,0\right)}$ with the second order ${\hat{T}}_{3}^{\left(1,0\right)}$, as in MRCCSD+T^*^(3), has been included (one iteration), to obtain the ${\hat{T}}_{3}^{\left(1,0\right)}$ values, which are correct up to the third order. The consequent ${\hat{H}}_{\mathrm{eff}}$ is correct at least up to the fourth order. This final approximation is known as MRCCSD+T^*^(4).

## 4. Bondonic Systematics of Electron Affinity Quantum Dynamics

Wherever the many electronic closed systems are present, starting with celebrating chemical bonding, special analysis and formalism should apply; this because the inter-electronic natural (Coulombic) repulsion acts as a potential barrier, preventing the inter-electronic interference, binding, and correlation. In fact, electrons as fermions interact through bosonic fields in the following way: in free states, the electromagnetic field is created, being quantified by the bosons—the photons; in *mutatis mutandis*, in closed or under externally applied potential systems, interacting electrons develop specific bosonic fields that drive their inter-relation dynamics. In chemical bonding, the descriptions of such bosonic fields were, by already a decade, quantified by the so-called bondons, the bosonic quasi-particle that is responsible for tunneling the inter-repulsion potential towards the electronic wave-function interference and bonding [74,75]. The bondonic formalism was soon recognized as “*…the bond is becoming again a central intellectual arena, and one can even find allusions to the bond as an elementary particle of chemistry, so-called <bondon>*” [76,77].

Accordingly, the bondonic theory introduces the needed chemical fields’ description of the chemical bond, though emphasizing the wave-particle duality, so filling the “missing link” of the celebrated molecular orbitals, density functional, and exclusively related wave-related theories [74,75]; this way, the chemical bonding is described as the bosonization of the interacting electrons, otherwise necessary repelling, through the quantum quasi-particles bosons, the bondons [75]. The bondons were considered to be the new challenge for the revived chemical boding arena [76,77], while, indeed, mainly addressing the exotic and complex features of atoms in molecules and the extended nano-chemical systems. The model proved to introduce an additional degree of freedom in interpreting chemical bonding, for instance, explaining why the ordinary (quantum) chemical bond is not possible in a helium molecule and, when possible, it relates through the bondons involved with the helium superfluidity [78]; it also offers the quantum counterpart of otherwise only purely topologically described Stone-Wales rotations’ formation and propagation on graphenes [79,80,81].

Thus, being a formalism for describing the interactions of many electronic systems, the bondonic formalism may also act as an independent, checking formalism for any other theory, as is the present coupled-cluster Fock-space-related one; even more, especially when it is about electronic affinity, the power of any theoretical description should also be formally checked by a dedicated theory, systematically allowing the tunneling of the inter-electronic repelling, with the affinity being the typical dynamical “adding electrons” to the “electronic bath”; this section is thus dedicated to pursue and prove that such connection can be formally established.

Quite recently, the bondonic formalism provided an alternative formal way of describing the fourth-order perturbation treatment of the ionization potentials of molecules, by advancing the *N*-body *k*-order of interaction of bondonic contribution to the specific contraction [71], as follows:

(17) B[1](α)×B[2](β)×B[3](γ)×11~N7⊗f(quantum−fluctuations)

with the following:

(18) B[N](k)=N22−(k+l)k1+k+11

*N*—the total number of electrons in the bonding state of matter (it can be either ground state or valence state, or another involved in chemical reactivity); *k*—the perturbation order; *l*—the total number of loops over all the diagrams involved [71,82].

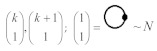

**Figure 1:**
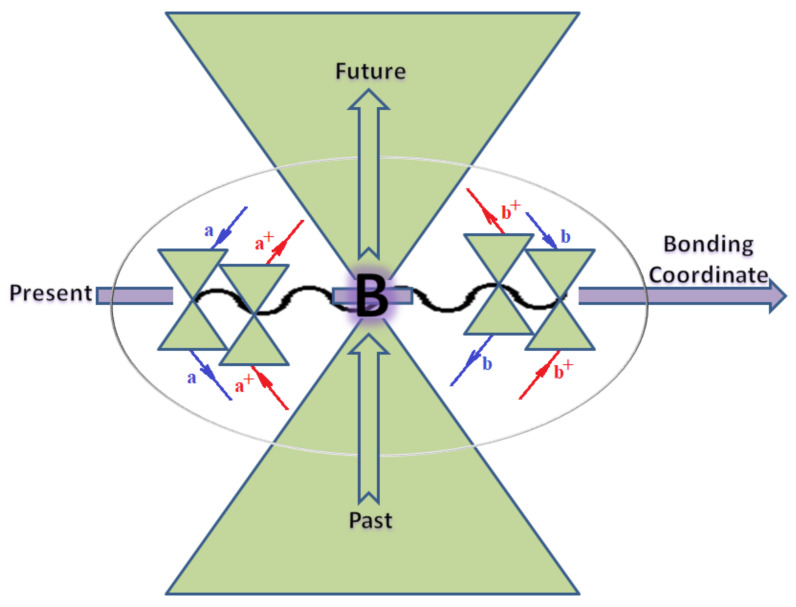
The Feynman’s bondonic prototype from creation and annihilation quantum operators; the resulting basic life-lines of electronic quantum dynamics structures include the following, from left to right: nucleophilic (active hole), electrophilic (active electron, conduction, ionization), radicalic affinity (active hole–electronic pair), and protonation (electronic annihilation in situ)—bosonizations, respectively. Basically, the entire chemical bonding and reactivity phenomenology can be described with the resulting “bondonic–zoo”; the respective classification, according to the relativistic-coupled (past-in and future-in bonding) spinning sectors of specific bondons, are in Table 1, systematically detailed—also see the text for further details.

Moreover, α,β,γ index the specific realization (viz. the orders), depending on the implemented scheme. Note that the quantum fluctuation function above may further affect the total order of collective electrons in bonding, depending on the bondonic creation–annihilation life-line of pairing electrons that contribute to bondonic bosons in their dynamics. Actually, in molecular ionization potential, many body schemes, such as fluctuation (creation vs. annihilation) along the line-life of electrons in affected bonds, was not explicitly considered, since the expulsed electrons is “certainly” observed or gained (i.e., no fluctuation). Yet, in general, and in the special present case of electronic affinity modeling, this will longer be possible to ignore—since the molecular virtual and excited orbitals are involved—so, this was included with necessity creation and annihilation dynamics in such complex perturbation quasi-particle quantum schemes. This way, Figure 1 depicts the “decomposed” Feynman diagram for bondonic types as they may appear through the creation and annihilation action of operators that are associated with the electrons in chemical bonding. Accordingly, the electrons in bonding interact not only in space—along the bonding coordinate—but also in time, by their creation and annihilation type of information/operators.

(19) $$\hat{o}=\left\{\begin{array}{l} 0\ldots \hat{a},\hat{b}\\ 1\ldots {\hat{a}}^{+},{\hat{b}}^{+} \end{array}\right.$$

They are naturally related with spinning too. Therefore, one may consider the basic “paste” and “future” spinning sectors as being of singlet and triplet forms, and with associated total spin quantum numbers $S_{\vee }$ and $S_{\vee }$, respectively, as follows:

(20) $$\vee ,\wedge :\left\{\begin{array}{l} T^{\pm }\ldots S_{\vee ,\wedge }=\pm 1\\ S\ldots S_{\vee ,\wedge }=0 \end{array}\right.$$

The resulting “present” chemical bonding contraction within the bondonic formation employs the “in-bonding” eigenvalues of creation–annihilation operators, in their possible pairings, as such the following applies:

(21) $$<,>:\left\{\begin{array}{l} \sum_{<}\hat{o}=-1,0,1,2\\ \sum_{>}\hat{o}=-1,0,1,2 \end{array}\right.$$

Chemically speaking, four types of in-bonding dynamics are actually combined, namely, the following: nucleophilic (active hole)—by past–future annihilation for electronic life-line in bonding; electrophilic (active electron, conduction, ionization)—by past–future creation for electronic life-line in bonding; radicalic affinity (active hole–electronic pair)—by past-annihilation future creation of electronic life-line in bonding; and protonation (in situ electronic annihilation)—by past-creation future annihilation of electronic life-line in bonding, respectively. The systematic coupling of these life-lines give out the corresponding special classes of bondons, as in Table 1, classified according to their past/future (spinning) sectors in bonding. At this point, it is worth mentioning that the ionization potential of the fourth-order scheme above, corresponds with employing the bondon B[N](k)=TT+/+[N](k), since it unitarily involves past–future active electrons in bonding towards the ionization level in molecular systems or to the conduction bands in solid systems. On the other hand, in the electronic affinity phenomenology that is associated with *active past hole–future electronic pairing*, the bondonic terms B[N](k)=TT*/*[N](k) are associated with their measure instead. However, in many electronic molecular systems, such electronic affinity bondonic terms turn out to appear not only by the single bosonization of paired interactions of electrons in bonding, but also through bondonic contraction by the paired interaction of bondons in the creation–annihilation dynamics across many electronic dynamics. Table 2 showcases all such diagrammatical contractions for each bondonic sector that is considered in Table 1. The emphasis (also by explicit depiction) is given on “exchanging-correlation” of life-lines of active hole–electronic pairings, resulting in electron affinity bondonic terms in various combinations, i.e., with the new distinctive notations, as follows:

(22) T**[N](k)=TT*/*

(23) T*−[N](k)=1TT−/−TS−/*TS*/−

(24) T*+[N](k)=1TT+/+ST+/*ST*/+

(25) T*0[N](k)=1TT0/0SS0/*SS*/0

Observe that the scheme for EA–bondonic T*+[N](k) explicitly depends on IP–bondonic $TT^{+/+}{\left[N\right]}^{\left(k\right)}$; this is also relevant for the present aim, in establishing which of the actual schemes may correspond to the Fock-space coupled-cluster approach, which states the fact the EA–bondons T*−[N](k) and T*+[N](k) are based on partial singlet partial triplet life-lines of electrons in bonding, so are naturally associated with the T*+[N](k) further partial scheme of perturbation. It is clear now that each such formal scheme of quantum electronic affinity, based on bondonic formalism, may be considered with the above general scheme of fourth-order bondonic expansion, giving rise to the actual specializations, which are conceptually equivalent with the Fock-space multi-reference coupled-cluster singles doubles and triplets (MRCCSD+T) methods for the interacting clusters of electrons, in an iteratively composed way.

(26) MRCCSD+T*(3)…T**[1](3)×T**[2](2)×T**[3](2)×11

(27) MRCCSD+T*(3)−a(4)… T*−[1](3)×T*−[2](3)×T*−[3](2)×11

(28) MRCCSD+T*(3)−b(4)…T*+[1](3)×T*+[2](2)×T*+[3](3)×11

(29) MRCCSD+T*(4)…T*0[1](3)×T*0[2](3)×T*0[3](3)×11

The final actual bondonic matter regards the possible generalization of the previously advanced form for the IP–bondonic perturbative expression; the general form is now proposed as follows:

(30) B[N](k)=TT+/+,TT−/−,TT0/0,TT*/*TS+/0,TS0/+,TS−/*,TS*/−ST+/*,ST0/−,ST*/+,ST−/0SS0/*,SS+/−,SS−/+,SS*/0=2−(k+l)−1N2N12∑<o^−1+N12∑>o^−1kS∨21+kS∧2+11

It nevertheless displays the following conceptually valid features: it naturally generalizes the *k*-order of interaction by the electronic spinning eigenvalue in bonding ($S_{\vee ,\wedge }^{2}$); it corrects the $N^{2}$ quadratic energy that is the generically assumed dependence in the bonding (via ionization and affinity averaging in bonding equilibrium) of adducts’ binding, such as the Parr approximation of atoms-in-molecule energy of bonding [83], to the actual quantum dynamics refinement, due to creation–annihilation life-line (contraction and superposition) of electrons/holes (numbers, also fractional, due to their quasi-particle/weaving nature) in bonding, by bondons. This way, the actual bondonic higher-order expansion also re-opens the E=E(N) general dependence in bonding, with direct conceptual consequences in the deeper understanding of chemical reactivity phenomenology, indices (especially electronegativity and chemical hardness), and their allied principles; however, it naturally recovers the IP–bondonic term once all the creation–annihilation operators and spinning sectors’ eigenvalues are respectively replaced with the actual entries for the $TT^{+/+}{\left[N\right]}^{\left(k\right)}$ term of Table 1.

## 5. Computational Details

The elements of the effective Hamiltonian ${\bar{f}}_{N},{\bar{v}}_{N},{\bar{w}}_{N}$ are obtained from contraction of the CC amplitudes ${\hat{T}}_{1}^{\left(0,0\right)}$ and ${\hat{T}}_{2}^{\left(0,0\right)}$, with Fock matrix elements $f_{i}^{a}$ and two-electron Slater integrals $v_{\mathrm{ij}}^{\mathrm{ab}}$. These are then stored as effective operators. These effective operators latter contract with the FSCC operators ${\hat{T}}_{1}^{\left(1,0\right)}$, ${\hat{T}}_{2}^{\left(1,0\right)}$, ${\hat{T}}_{3}^{\left(1,0\right)}$, to provide the contribution to the FSCCSD+(T) diagrams. We have rearranged the loop structure to maintain the N^7^ power algorithm.

For the test of the theory, we have taken three examples ${\mathrm{Li}}_{2}$, BeO, and CH^+^. For small molecules, the calculation of electron affinity of these molecules is a challenge. For ${\mathrm{Li}}_{2}$, we have calculated both the vertical electron affinity (VEA) at the experimental inter-nuclear distance 5.051 a.u., and the adiabatic electron affinity (AEA) of ${\mathrm{Li}}_{2}$, for which the ground-state geometry of ${\mathrm{Li}}_{2}^{-}$ (6.0 a.u. inter-nuclear distance) is used. We have used four different basis sets, A, B, C and D, as will be explained later in this section. Since we are interested in the lowest electron affinity of the di-lithium molecule, we have reported only one value. However, in two of the bases, basis-B and -D, as will be explained later, the four particles are reasonably close-lying and are treated as active particles. In the case of two other bases—A and C—we have taken only one active particle. In these two cases, the separation of this one active particle from the rest of what is termed inactive subspace is sufficient not to warrant any convergence problem. In the cases of four active particles, due to the symmetry, the effective Hamiltonian turned out to be diagonal. It may be mentioned that in our code, we have not used symmetry explicitly.

To obtain AEA of ${\mathrm{Li}}_{2}$, we first calculate the vertical electron affinity of Li_2_ at the geometry of Li_2_^−^, using a similar number of active particles. These electron affinities can be called the vertical ionization of ${\mathrm{Li}}_{2}^{-}$, within the Franck–Condon envelope of the anion ground-state geometry. These are reported as the vertical ionization potentials of ${\mathrm{Li}}_{2}^{-}$. Then, by subtracting these from the ground-state energy of ${\mathrm{Li}}_{2}^{-}$ at the ${\mathrm{Li}}_{2}^{-}$ geometry, we obtain the total energy of ${\mathrm{Li}}_{2}^{-}$ in several schemes. Finally, subtracting from the ground-state energy of ${\mathrm{Li}}_{2}$ at the neutral ${\mathrm{Li}}_{2}$ geometry, AEA of ${\mathrm{Li}}_{2}$ is obtained. The adiabatic electron affinity calculation is thus no longer a direct calculation.

The basis sets that are used for the calculations are of DZVP and TZVP quality, based on Dunning’s correlation consistent basis set in the absence and presence of diffuse functions. The basis sets are constructed starting with a polarized split-valence basis set, obtained from the Gamess-US package. This basis-A (3s2p1d) is further improved by the addition of an extra set of diffuse functions [84], without altering the core and valence shell exponents, leading to a 4s3p1d basis set (basis-B). The need for extra diffuse s and p functions to the existing basis set is important for calculations of electron affinity. The basis-C (4s3p1d) is just a TZVP-type basis set and the addition of an extra set of diffuse functions to the basis results in basis-D (5s4p1d). The detailed basis sets have been presented in the Supplementary Materials (SI-1). An extensive discussion of the results, with the inclusion of different schemes of triples and differing bases sets, has been presented in the next section.

As the next example, we have calculated the vertical electron affinity of BeO. It is well known that the determination of the electron affinity of the BeO molecule is quite difficult, due to the significant mobility of the electronic charge for BeO along the bond axis, due to the 2s-2p degeneracy of Be. The RHF of BeO is the ^1^∑ determinant $\left|1\sigma^{2}2\sigma^{2}3\sigma^{2}4\sigma^{2}1\Pi^{4}\right|$. The LUMO of BeO is 5σ and the next unoccupied orbital 2Π is quite close. Hence, due to the quasi-degeneracy, we have included three active particles, consisting of σ and Π symmetry (5σ and 2Π). However, what is of relevance is the electron affinity resulting from the electron attachment in the LUMO of the σ symmetry (i.e., 5σ orbital). Thus, BeO is a prototype example of a difficult molecule, where the multi-determinant character of the reference is required. This allows a more extensive test of the code generated. Due to the symmetry, the three-dimensional effective Hamiltonian is diagonal, with the diagonal elements corresponding to the two states corresponding to Π being equal. The calculations are conducted at the Be–O bond distance of 2.515 a.u., using two basis sets. The basis sets used are of standard DZVP and aug-cc-pVDZ quality. The results are presented, analyzing the importance of triples as well as basis sets.

As a final example, we have presented the VEA of CH^+^ at the equilibrium geometry of the CH radical, i.e., 1.129 a.u. The motivation of carrying out the calculations at the equilibrium geometry of the CH radical is to compare this against the experimental vertical ionization potential of the CH radical. The four different basis sets have been used. Basis-A consists of the cc-PVDZ basis, and basis-B is the aug-cc-PVDZ basis. Basis-C and D are the cc-PVTZ and aug-cc-PVTZ bases, respectively. We used all three holes of ${\mathrm{CH}}^{+}$ as active, but present the lowest EA in the table, which turns out to be the lowest IP of the CH radical.

## 6. Results and Discussion

First, we present the result of the EA of ${\mathrm{Li}}_{2}$ in the different schemes proposed. The result presented is that of the state corresponding to the only bound state of the Li_2_ anion.

Since the anion has the geometry of 6.0 a.u., the adiabatic calculations involved the difference of energy at the ${\mathrm{Li}}_{2}^{-}$ geometry of 6.0 a.u., and that at the neutral Li_2_ geometry. The adiabatic ones are expected to be closer to the experimental geometry.

For comparison, the adiabatic experimental EA is 0.437 ± 0.009 eV [84] and an earlier reported theoretical calculation provided a result of 0.90 eV [85]. Table 3 presents the vertical electron affinity of Li_2_. In the smallest basis-A, which is of just DZVP quality, using one active particle, we observe that the FSMRCCSD calculation underestimates the electron affinity. Triples at the third order do not improve the results, as reflected by the $T^{*}\left(3\right)$ results. A more significant change takes place at the $T^{*}-a\left(4\right)$ level, where ${\hat{T}}_{2}^{\left(1,0\right)}$ amplitudes are corrected at the third order, due to second-order triples, and the resulting effective Hamiltonian gets corrected at the fourth order. The result changes the electron affinity towards the experiment. At the $T^{*}-b\left(4\right)$ and $T^{*}\left(4\right)$ level, the results oscillate. It is gratifying to note the improvement of the result at different fourth-order schemes, compared to third-order triples, as well as FSMRCCSD. We can only expect further improvement where the adiabatic electron affinity is presented. It is noteworthy to observe that basis-A is the smallest of the bases and does not contain any diffuse functions.

If we turn our attention to basis-B, which includes a diffuse set of functions on to basis-A, we observe an improvement in the results. The calculations are reported using four active particles. Due to the symmetry of the active particles, the effective Hamiltonian is fully diagonal, as mentioned earlier, and it is gratifying to note that the code, without explicit use of symmetry, reproduces that. As highlighted in the earlier section, however, we present the result of only one state, which corresponds to the lowest bound Li_2_ anion. The trends of different theoretical schemes are similar, in that the significant improvement takes place at the $T^{*}-a\left(4\right)$ level itself. As expected, the diffuse functions improve the results significantly. For basis-C, which includes only extended valence functions in addition to basis-A, we have again taken only one active particle, for the same reason as in the case of basis-A. The general trends, in terms of theory for the EA presented, are again the same. There is an improvement compared to basis-A, but the values decrease compared to basis-B, showing that the diffuse functions are more important than the valence functions.

Basis-D includes both additional valence and diffuse functions, and thus is the most extensive basis used for the example molecule. As in basis-B, we have taken four active particles, since the addition of diffuse functions in the basis brings the first four particles closer. We have reported one relevant EA in this case too. The effective Hamiltonian, as expected, is completely diagonal. We find similar trends of results in different theoretical approximations, highlighting the importance of fourth-order triples. The results are even better than either basis-B or -C, which is to be expected. All these are expected to increase further for adiabatic calculations, bringing them closer to the experimental value. The effects of diffuse functions, as well as additional valence sets, improve the EA. Both these additional basis functions stabilized the neutral and anion molecules, though the stabilization is more pronounced for the anion, resulting in an increase in EA. Among these, the extra diffuse set of functions plays a more important role.

In Table 4, we have presented the vertical IP of ${\mathrm{Li}}_{2}^{-}$ in the four bases. This distance of ${\mathrm{Li}}_{2}^{-}$ is larger than that of ${\mathrm{Li}}_{2}$. These are calculated as the vertical EA of ${\mathrm{Li}}_{2}$ at the geometry of ${\mathrm{Li}}_{2}^{-}$. The number of active particles that is considered is the same as in the case of the vertical EA calculation in each of the four cases. In basis-B and -D, the effective Hamiltonian turns out to be completely diagonal. The general trends are similar. Compared to the CCSD level, the $T^{*}\left(3\right)$ has no significant change. The major improvement takes place at the $T^{*}-a\left(4\right)$ level in all the bases. Using these results and the ground-state energy of ${\mathrm{Li}}_{2}$ at the CCSD level, at the geometry of ${\mathrm{Li}}_{2}\;\mathrm{and}\;{\mathrm{Li}}_{2}^{-}$, we can calculate the adiabatic EA, which is presented in Table 5.

The general trends are similar and fourth-order correction turns out to be very important. One can observe the results closer to the experiment. In the most extensive basis, i.e., basis-D, the adiabatic EA turns out to be 0.365 eV, which is very close to the experimental value of about 0.437 ± 0.009 eV.

In Table 6, we present the results of the vertical electron affinity of BeO, only for the 5σ orbital, resulting in, out of three active particles, 5σ and 2π. We present the results in the DZVP and aug-cc-pVDZ basis. In each of the bases, the effective Hamiltonian matrix turns out to be diagonal, as is expected, due to the symmetry. We, however, report the only state for which there is a positive EA. Here, we find that, compared to the experimental value, the effect of triples fails to improve the results. However, as soon as these are computed using fourth-order schemes, we note the improvement towards the experiment. It highlights the importance of fourth-order triples, compared to the third order. In particular, we observe that in the aug-cc-pVDZ basis, which includes diffuse functions, the results are remarkably close to the experimental value, keeping in view that this is the vertical electron affinity calculation. We have also reported CCSD(T) values, which are very accurate results with triples. We observe that our results at the $T^{*}-a\left(4\right)$ and $T^{*}-b\left(4\right)$ level are quite close to the full T^*^(4) results, as well as the CCSD (T) results, in each of the bases, and in the more extensive basis the result is indeed remarkably close to the experiment [84,86].

Table 7 presents the results of the vertical electron affinity of ${\mathrm{CH}}^{+}$, or the vertical ionization potential of the CH radical. The lowest ionization potential of the CH radical is reported under the experiment column at the experimental bond length of 1.129 a.u. This is an interesting application, where the ionization potential of a radical is computed as the electron affinity of the corresponding cation. Five different methods, one with MRCCSD, and four other triples-corrected ones, are presented. What is relevant for this paper is the three partial and full fourth-order triples-corrected schemes. The trends of the methods are similar in each case. From $T^{*}\left(3\right)$, to $T^{*}-a\left(4\right)$, the values increase. At the $T^{*}-b\left(4\right)$ level, the result decreases, which is followed by a marginal decrease at the full $T^{*}\left(4\right)$ level. What has transpired from these is the fact that the $T^{*}-b\left(4\right)$ level results are nearly the same as the full $T^{*}\left(4\right)$ results. Obviously, these partial fourth-order results take less computing time, even at the $T^{*}-a\left(4\right)$ level, and the results are quite satisfactory. One observes the effect of diffuse function as one that is evident from the results of basis-A to basis-B, and similarly from basis-C to basis-D. Even with an oscillating trend, the main outcome is the efficacy of the partial fourth-order schemes, as in the previous cases.

## 7. Computational Cost

We now look at the computational cost of different fourth-order schemes. We note that the schemes of third-order and fourth-order triples are calculated in the order MRCCSD+T^*^(3), MRCCSD+T^*^−a(4), MRCCSD+T^*^−b(4), and MRCCSD+T^*^(4). All of these scale as N^7^. However, third-order triples are calculated first and the equations contain very few diagrams. With a low prefactor, these calculations are fast. The next scheme computed is T^*^−a(4), followed by T^*^−b(4), and finally the full fourth order. Clearly, the computational times required go up in the same order. However, it is important to note that, in terms of diagrams, the two latter schemes T^*^−b(4) and the full fourth order, have more in number and thus contain a larger prefactor. The typical computing time to calculate T^*^(4) is about 4 times the time that is taken for T^*^−a(4), for the calculations of EA for the molecules that we presented. The codes are not parallelized, and thus the exact computing times are not of much interest. The prefactor for the T^*^−a(4) scheme is much smaller compared to the prefactor for T^*^−b(4). From the point of view of the reliability of the numbers vis-à-vis the computational time, the T^*^−a(4) scheme is a promising candidate for the approximate inclusion of fourth-order triples to EA calculation.

## 8. Conclusions

The approximate triples models of the Fock-space coupled-cluster method, MRCCSD+$T^{*}\left(3\right)$, partially corrected the fourth-order schemes MRCCSD+T^*^−a(4) and MRCCSD+T^*^−b(4), and fully corrected MRCCSD+T^*^(4) schemes have been presented for direct EA calculation, based on the perturbative inclusion of triples on the full singles and doubles model. The EA calculation can be used for IP calculations of the corresponding anion. Model calculations of EA on ${\mathrm{Li}}_{2}$, BeO, and CH^+^ in different basis sets have been presented. The effects of diffuse and valence basis sets have been analyzed. For all the cases, we find that the diffuse functions improve the EA. It is important to stress that in all the cases, the partial triples at third order do not improve the results. However, different schemes of partial fourth order offer improvements and are in significant agreement with the experiment. The a(4) scheme is the computationally least expensive, followed by the b(4) and full scheme. What is instructive is to note that a(4) itself provides results that are quite reliable. The adiabatic EA calculations for Li_2_ are, as expected, even closer to the experiment [84]. In the case of Li_2_, we also note significant improvement of the earlier theoretical calculation [89], which reports a value of 0.90 eV for adiabatic EA, which is higher than the experimental EA. To conclude, it appears that these partial fourth-order schemes of FSMRCC methods are extremely promising approaches for EA calculations, while the newly emerging bondonic formalism also successfully encompassed the affinity effects, yet in a more coupling complexification, respecting the counterpart earlier ionization framework.

## Figures and Tables

**Table 1:** Bondonic Feynman’s-type diagrams for the triplet (T), singlet (S) and mixed sectors of creation–annihilation life-lines of creation–annihilation of valence electrons in generic chemical bonding; the corresponding left (<) and right (>) time-ordering contraction for the operatorial eigenvalues are respectively considered relative with the “present” in or out electrons in bonding (see text for further details); the resulting total spin for past (∨) and future (∧) “histories” of in and out valence electrons in bonding are also considered and correlated with the triplet and singlet total spin in each of TT, SS, ST and TS bondonic sectors, respectively (see also Figure 1 for details on symbolic representation of bondons).

Bondonic Diagram	Bondonic Symbol	Quantum Indices			
		*Bonding Life-Lines of* *Creation–Annihilation*		*Total* *Spin*	
		$\pm \sum_{<}\hat{o}$	$\pm \sum_{>}\hat{o}$	$S_{\vee }$	$S_{\wedge }$
“TT” Sector					
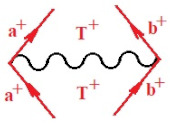	TT^+/+^	2	2	1	1
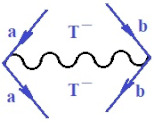	TT^−/−^	0	0	−1	−1
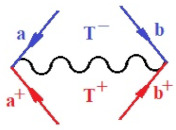	TT^0/0^	−1	−1	1	−1
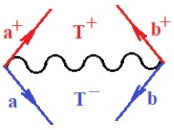	TT^*/*^	+1	+1	−1	1
“TS” Sector					
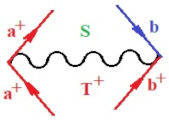	TS^+/0^	2	−1	1	0
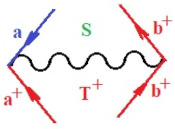	TS^0/+^	−1	2	1	0
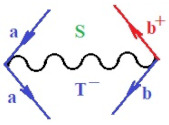	TS^−/*^	0	+1	−1	0
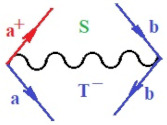	TS^*/−^	+1	0	−1	0
“ST” Sector					
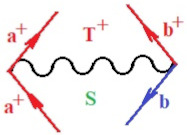	ST^+/*^	2	1	0	1
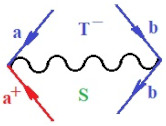	ST^0/−^	−1	0	0	−1
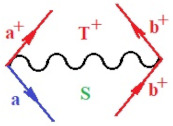	ST^*/+^	+1	2	0	1
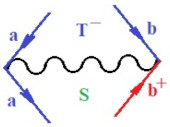	ST^−/0^	0	−1	0	−1
“SS” Sector					
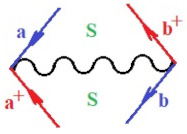	SS^0/*^	−1	+1	0	0
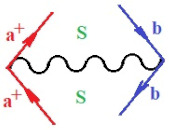	SS^+/−^	2	0	0	0
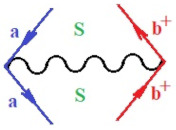	SS^−/+^	0	2	0	0
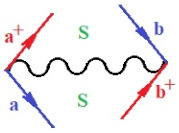	SS^*/0^	+1	−1	0	0

**Table 2:** Bondonic contractions along the reaction coordinates, while involving the left and right creation–annihilation life-lines for all combinations specific to each “*past-future*” triplet and singlet total spin sectors of corresponding Feynman’s diagrams for the “present” in and out valence electrons in a generic chemical bonding and reaction mechanism; special emphasis by detailed Feynman bondonic diagrammatic contraction is given for those interactions involving/resulting in TT^*/*^ product, for each bondonic sector, as such terms are currently associated with the electronic affinity bonding/bondonic contribution (see text for further details and Figure 1 for more on symbolic and diagrammatic representation of bondons).

BONDONS-IN				BONDONS-OUT			
Term “p”	⊗	Term “q”	=		Term “r”	⊗	Term “w”
“TT” Sector							
TT^+/+^	⊗	TT^−/−^	=		SS^−/+^	⊗	SS^+/−^
TT^+/+^	⊗	TT^0/0^	=		TS^0/+^	⊗	TS^+/0^
TT^+/+^	⊗	TT^*/*^	=		ST^*/+^	⊗	ST^+/*^
TT^−/−^	⊗	TT^0/0^	=		ST^0/−^	⊗	ST^−/0^
TT^−/−^	⊗	TT^*/*^	=		TS^*/−^	⊗	TS^−/*^
TT^0/0^	⊗	TT^*/*^	=		SS^*/0^	⊗	SS^0/*^
“TS” Sector							
TS^+/0^	⊗	TS^0/+^	=		TT^0/0^	⊗	TT^+/+^
TS^+/0^	⊗	TS^−/*^	=		ST^−/0^	⊗	ST^+/*^
TS^+/0^	⊗	TS^*/−^	=		SS^*/0^	⊗	SS^+/−^
TS^0/+^	⊗	TS^−/*^	=		SS^−/+^	⊗	SS^0/*^
TS^0/+^	⊗	TS^*/−^	=		ST^+/+^	⊗	ST^0/−^
TS^−/*^	⊗	TS^*/−^	=		TT^*/*^	⊗	TT^−/−^
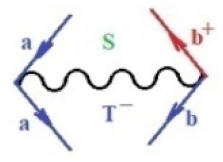	⊗	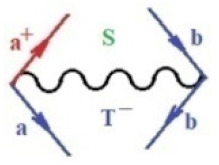	=		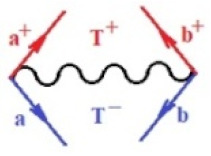	⊗	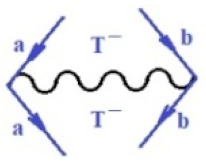
“ST” Sector							
ST^−/0^	⊗	ST^0/−^	=		TT^0/0^	⊗	TT^−/−^
ST^−/0^	⊗	ST^+/*^	=		TS^+/0^	⊗	TS^−/*^
ST^−/0^	⊗	ST^*/+^	=		SS^+/0^	⊗	SS^−/+^
ST^0/−^	⊗	ST^+/*^	=		SS^+/−^	⊗	SS^0/*^
ST^0/−^	⊗	ST^*/+^	=		TS^+/−^	⊗	TS^0/+^
ST^+/*^	⊗	ST^*/+^	=		TT^*/*^	⊗	TT^+/+^
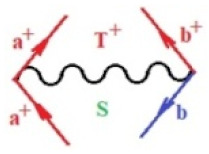	⊗	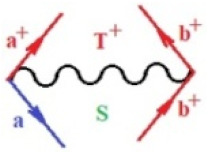	=		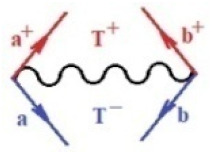	⊗	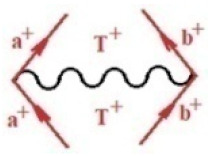
“SS” Sector							
SS^+/−^	⊗	SS^−/+^	=		TT^−/−^	⊗	TT^+/+^
SS^+/−^	⊗	SS^0/*^	=		ST^0/−^	⊗	ST^+/*^
SS^+/−^	⊗	SS^*/0^	=		TS^*/−^	⊗	TS^+/0^
SS^−/+^	⊗	SS^0/*^	=		TS^0/+^	⊗	TS^−/*^
SS^−/+^	⊗	SS^*/0^	=		ST^*/+^	⊗	ST^−/0^
SS^0/*^	⊗	SS^*/0^	=		TT^*/*^	⊗	TT^0/0^
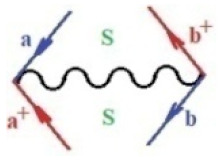	⊗	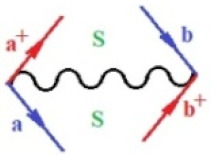	=		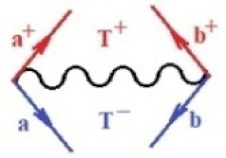	⊗	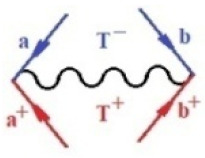

**Table 3:** Vertical electron affinity of ${\mathrm{Li}}_{2}$ using various basis sets at inter-nuclear separation of 5.05 a. u.

Methods	Results (eV)			
	Basis-A (3s2p1d)	Basis-B(4s3p1d)	Basis-C(4s3p1d)	Basis-D(5s4p1d)
MRCCSD	0.201	0.268	0.241	0.279
MRCCSD+T^*^(3)	0.190	0.254	0.228	0.265
MRCCSD+T^*^−a(4)	0.279	0.316	0.305	0.329
MRCCSD+T^*^−b(4)	0.246	0.290	0.274	0.303
MRCCSD+T^*^b(4)	0.253	0.294	0.280	0.307
Experimental [84,86]	0.437 ± 0.009			

**Table 4:** Vertical ionization potential of ${\mathrm{Li}}_{2}^{-}$ using various basis sets at inter-nuclear separation of 6.0 a. u.

Methods	Results (eV)			
	Basis-A (3s2p1d)	Basis-B (4s3p1d)	Basis-C (4s3p1d)	Basis-D (5s4p1d)
MRCCSD	0.356	0.421	0.399	0.435
MRCCSD+T^*^(3)	0.358	0.416	0.399	0.430
MRCCSD+T^*^−a(4)	0.455	0.499	0.491	0.515
MRCCSD+T^*^−b(4)	0.424	0.472	0.461	0.488
MRCCSD+T^*^b(4)	0.436	0.481	0.472	0.497

**Table 5:** Total energy of ${\mathrm{Li}}_{2}^{-}$ in a.u.and adiabatic electron affinity of ${\mathrm{Li}}_{2}$ in eV using various basis sets at inter-nuclear separation of 6.0 a. u.

Methods	Basis-A (3s2p1d)	Basis-B (4s3p1d)	Basis-C (4s3p1d)	Basis-D (5s4p1d)
	Total Energy of Li_2_^−^ (a.u.)			
MRCCSD	−14.9122	−14.9148	−14.9415	−14.9430
MRCCSD+T^*^(3)	−14.9123	−14.9147	−14.9415	−14.9428
MRCCSD+T^*^−a(4)	−14.9159	−14.9177	−14.9449	−14.9459
MRCCSD+T^*^−b(4)	−14.9147	−14.9167	−14.9438	−14.9449
MRCCSD+T^*^b(4)	−14.9152	−14.9171	−14.9442	−14.9452
	Results (eV)			
MRCCSD	0.225	0.291	0.267	0.303
MRCCSD+T^*^(3)	0.228	0.286	0.267	0.298
MRCCSD+T^*^−a(4)	0.324	0.369	0.360	0.384
MRCCSD+T^*^−b(4)	0.293	0.342	0.329	0.357
MRCCSD+T^*^b(4)	0.306	0.351	0.340	0.365
Experimental [84,86]	0.437 ± 0.009			

**Table 6:** Vertical electron affinity of BeO using various basis sets at inter-nuclear separation of 2.515 a. u.

Methods	Results (eV)	
	Basis-I	Basis-II
MRCCSD	1.81	2.05
MRCCSD+T^*^(3)	1.66	1.92
MRCCSD+T^*^−a(4)	1.81	2.09
MRCCSD+T^*^−b(4)	1.80	2.07
MRCCSD+T^*^b(4)	1.84	2.12
CCSD (T) [87,88]	1.79	2.18
Experimental [84,86]	2.15 ± 0.05	

**Table 7:** Vertical electron affinity of ${\mathrm{CH}}^{+}$ using various basis sets at inter-nuclear separation of 1.12 a. u.

Methods	Results (eV)			
	Basis-A	Basis-B	Basis-C	Basis-D
MRCCSD	10.307	10.528	10.409	10.564
MRCCSD+T^*^(3)	10.217	10.416	10.321	10.450
MRCCSD+T^*^−a(4)	10.401	10.706	10.508	10.722
MRCCSD+T^*^−b(4)	10.382	10.680	10.487	10.696
MRCCSD+T^*^b(4)	10.366	10.675	10.474	10.691
Experimental [84,86]	10.64			

## Data Availability

Supplementary Materials  available.

## Supplementary Materials

The following are available online at https://www.mdpi.com/article/10.3390/ijms22168953/s1, Supplementary Materials (SI-1) contains the information on four basis sets, A, B, C, D (as referred in the Section 5) of Li_2_ that have been used to obtain vertical EA.
