# Supplementary material for: Three-Body Excitations in Fock-Space Coupled-Cluster: Fourth Order Perturbation Correction to Electron Affinity and Its Relation to Bondonic Formalism
Source: Int J Mol Sci. 2021 Aug 19;22(16):8953. doi: 10.3390/ijms22168953 (PMC8396530; doi:10.3390/ijms22168953)
Supplement: Supplementary file 1 [file ijms-22-08953-s001.zip › ijms-1338698-supplementary.pdf]

Supplementary Information (SI-1)

Three-Body Excitations in Fock-Space Coupled-Cluster: Fourth Order Perturbation Correction to Electron Affinity and Its Relation to Bondonic Formalism

Suhita Basumallick [1], Mihai V. Putz [2,*] and Sourav Pal [1,3,*]

[1] Department of Chemistry, Indian Institute of Technology Bombay, Powai, Mumbai 400076, India; basumallick.suhita16@gmail.com
[2] Laboratory of Structural and Computational Physical-Chemistry for Nanosciences and QSAR, Biology-Chemistry Department, Faculty of Chemistry, Biology, Geography, West University of Timisoara, Str. Pestalozzi No. 16, 300115 Timisoara, Romania
[3] Department of Chemical Sciences, Indian Institute of Science Education and Research Kolkata, Mohanpur, Nadia 741246, India
* Correspondence: mihai.putz@e-uvt.ro (M.V.P.); s.pal@iiserkol.ac.in (S.P.); Tel.: +40-0256-592-631 (M.V.P.); +91-33-6136-0012 (S.P.)

A.1Di-Lithium Molecule (Li_2): at internuclear distance 5.05 a.u

Basis-A

```
li        3.0       0.0000         0.0000     0.0000
S   8
  1  1469.0000000          0.000765601232
  1   220.5000000          0.005888932715
  1    50.2600000          0.029655553732
  1    14.2400000          0.109123162563
  1     4.5810000          0.282641784375
  1     1.5800000          0.452887111102
  1     0.5640000          0.274630956861
  1     0.0734500          0.009745923779
S   8
  2  1469.0000000        -0.000224991961
  2   220.5000000        -0.001730563167
  2    50.2600000        -0.008791560880
  2    14.2400000        -0.033152565467
  2     4.5810000        -0.091687974012
  2     1.5800000        -0.180010443274
```

```
  2      0.5640000          -0.255703363785
  2      0.0734500           1.078277723416
S   1
  3      0.0280500           1.000000000000
P   3
  4      1.5340000           0.038003981035
  4      0.2749000           0.232032118583
  4      0.0736200           0.834631408456
P   1
  5      0.0240300           1.000000000000
D   1
  6      0.1239000           1.000000000000

li        3.0       0.0000         0.0000         5.0512375652
S   8
7  1469.0000000         0.000765601232
  7   220.5000000          0.005888932715
  7    50.2600000          0.029655553732
  7    14.2400000          0.109123162563
  7     4.5810000          0.282641784375
  7     1.5800000          0.452887111102
  7     0.5640000          0.274630956861
  7     0.0734500          0.009745923779
S   8
8  1469.0000000        -0.000224991961
  8   220.5000000         -0.001730563167
  8    50.2600000         -0.008791560880
  8    14.2400000         -0.033152565467
  8     4.5810000         -0.091687974012
  8     1.5800000         -0.180010443274
  8     0.5640000         -0.255703363785
  8     0.0734500          1.078277723416
S   1
  9     0.0280500          1.000000000000
P   3
  10    1.5340000          0.038003981035
  10    0.2749000          0.232032118583
  10    0.0736200          0.834631408456
P   1
  11    0.0240300          1.000000000000
D   1
  12    0.1239000          1.000000000000
```

Basis-B

```
li       3.0          0.0000             0.0000           0.0000
S   8
1  1469.0000000          0.000765601232
  1   220.5000000           0.005888932715
```

```
  1    50.2600000           0.029655553732
  1    14.2400000           0.109123162563
  1     4.5810000           0.282641784375
  1     1.5800000           0.452887111102
  1     0.5640000           0.274630956861
  1     0.0734500           0.009745923779
S   8
2  1469.0000000        -0.000224991961
  2   220.5000000          -0.001730563167
  2    50.2600000          -0.008791560880
  2    14.2400000          -0.033152565467
  2     4.5810000          -0.091687974012
  2     1.5800000          -0.180010443274
  2     0.5640000          -0.255703363785
  2     0.0734500           1.078277723416
S   1
  3     0.0280500           1.000000000000
S   1
  4     0.0074000           1.000000000000
P   3
  5     1.5340000           0.038003981035
  5     0.2749000           0.232032118583
  5     0.0736200           0.834631408456
P   1
  6     0.0240300           1.000000000000
P   1
  7     0.0074000           1.000000000000
D   1
  8     0.1239000           1.000000000000

li      3.0       0.0000           0.0000         5.0512375652
S   8
9  1469.0000000         0.000765601232
  9   220.5000000           0.005888932715
  9    50.2600000           0.029655553732
  9    14.2400000           0.109123162563
  9     4.5810000           0.282641784375
  9     1.5800000           0.452887111102
  9     0.5640000           0.274630956861
  9     0.0734500           0.009745923779
S   8
10  1469.0000000        -0.000224991961
  10   220.5000000          -0.001730563167
  10    50.2600000          -0.008791560880
  10    14.2400000          -0.033152565467
  10     4.5810000          -0.091687974012
  10     1.5800000          -0.180010443274
  10     0.5640000          -0.255703363785
  10     0.0734500           1.078277723416
S   1
  11     0.0280500           1.000000000000
```

```
S   1
  12     0.0074000          1.000000000000
P   3
  13    1.5340000          0.038003981035
  13    0.2749000          0.232032118583
  13    0.0736200          0.834631408456
P   1
  14    0.0240300          1.000000000000
P   1
  15    0.0074000          1.000000000000
D   1
  16    0.1239000          1.000000000000
```

Basis-C

```
li      3.0        0.0000           0.0000           0.0000
S   9
1  5988.0000000          0.000133195806
  1   898.9000000          0.001026509035
  1   205.9000000          0.005279761591
  1    59.2400000          0.020959812281
  1    19.8700000          0.066437667674
  1     7.4060000          0.166019058768
  1     2.9300000          0.315501808091
  1     1.1890000          0.394102355987
  1     0.4798000          0.191151004356
S   9
2  5988.0000000         -0.000077656259
  2   898.9000000         -0.000595364655
  2   205.9000000         -0.003032292029
  2    59.2400000         -0.012299272305
  2    19.8700000         -0.038898390070
  2     7.4060000         -0.103900377014
  2     2.9300000         -0.206846691413
  2     1.1890000         -0.366970200153
  2     0.4798000         -0.414865622550
S   1
  3     0.0750900          1.000000000000
S   1
  4     0.0283200          1.000000000000
P   3
  5     3.2660000          0.035544688557
  5     0.6511000          0.195796454763
  5     0.1696000          0.863995412271
P   1
  6     0.0557800          1.000000000000
P   1
  7     0.0205000          1.000000000000
D   1
  8     0.1239000          1.000000000000
```

```
li       3.0         0.0000          0.0000       5.0512375652
S   9
  9   5988.0000000          0.000133195806
  9    898.9000000          0.001026509035
  9    205.9000000          0.005279761591
  9     59.2400000          0.020959812281
  9     19.8700000          0.066437667674
  9      7.4060000          0.166019058768
  9      2.9300000          0.315501808091
  9      1.1890000          0.394102355987
  9      0.4798000          0.191151004356
S   9
10  5988.0000000         -0.000077656259
  10   898.9000000          -0.000595364655
  10   205.9000000          -0.003032292029
  10    59.2400000          -0.012299272305
  10    19.8700000          -0.038898390070
  10     7.4060000          -0.103900377014
  10     2.9300000          -0.206846691413
  10     1.1890000          -0.366970200153
  10     0.4798000          -0.414865622550
S   1
  11     0.0750900          1.000000000000
S   1
  12     0.0283200          1.000000000000
P   3
  13     3.2660000          0.035544688557
  13     0.6511000          0.195796454763
  13     0.1696000          0.863995412271
P   1
  14     0.0557800          1.000000000000
P   1
  15     0.0205000          1.000000000000
D   1
  16    0.1239000           1.000000000000
```

Basis-D

```
li       3.0          0.0000            0.0000          0.0000
S   9
1  5988.0000000           0.000133195806
  1   898.9000000           0.001026509035
  1   205.9000000           0.005279761591
  1    59.2400000           0.020959812281
  1    19.8700000           0.066437667674
  1     7.4060000           0.166019058768
  1     2.9300000           0.315501808091
  1     1.1890000           0.394102355987
  1     0.4798000           0.191151004356
```

```
S   9
2  5988.0000000          -0.000077656259
  2   898.9000000          -0.000595364655
  2   205.9000000          -0.003032292029
  2    59.2400000          -0.012299272305
  2    19.8700000          -0.038898390070
  2     7.4060000          -0.103900377014
  2     2.9300000          -0.206846691413
  2     1.1890000          -0.366970200153
  2     0.4798000          -0.414865622550
S   1
  3     0.0750900           1.000000000000
S   1
  4     0.0283200           1.000000000000
S   1
  5     0.0074000           1.000000000000
P   3
  6     3.2660000           0.035544688557
  6     0.6511000           0.195796454763
  6     0.1696000           0.863995412271
P   1
  7     0.0557800           1.000000000000
P   1
  8     0.0205000           1.000000000000
P   1
  9     0.0074000           1.000000000000
D   1
  10     0.1239000           1.000000000000

li       3.0       0.0000         0.0000         5.0512375652
S   9
  11   5988.0000000          0.000133195806
  11    898.9000000          0.001026509035
  11    205.9000000          0.005279761591
  11     59.2400000          0.020959812281
  11     19.8700000          0.066437667674
  11      7.4060000          0.166019058768
  11      2.9300000          0.315501808091
  11      1.1890000          0.394102355987
  11      0.4798000          0.191151004356
S   9
12  5988.0000000          -0.000077656259
  12   898.9000000          -0.000595364655
  12   205.9000000          -0.003032292029
  12    59.2400000          -0.012299272305
  10    19.8700000          -0.038898390070
  10     7.4060000          -0.103900377014
  10     2.9300000          -0.206846691413
  12     1.1890000          -0.366970200153
  12     0.4798000          -0.414865622550
S   1
```

```
  13      0.0750900          1.000000000000
S   1
  14      0.0283200          1.000000000000
S   1
  15      0.0074000          1.000000000000
P   3
  16      3.2660000          0.035544688557
  16      0.6511000          0.195796454763
  16      0.1696000          0.863995412271
P   1
  17      0.0557800          1.000000000000
P   1
  18      0.0205000          1.000000000000
P   1
  19      0.0074000          1.000000000000
D   1
  20    0.1239000            1.000000000000
```

A.2 Di-Lithium Molecule (Li_2): at internuclear distance 6.0 a.u

Basis-A

```
li       3.0         0.0000             0.0000          0.0000
S   8
1  1469.0000000           0.000765601232
  1   220.5000000           0.005888932715
  1    50.2600000           0.029655553732
  1    14.2400000           0.109123162563
  1     4.5810000           0.282641784375
  1     1.5800000           0.452887111102
  1     0.5640000           0.274630956861
  1     0.0734500           0.009745923779
S   8
2  1469.0000000         -0.000224991961
  2   220.5000000         -0.001730563167
  2    50.2600000         -0.008791560880
  2    14.2400000         -0.033152565467
  2     4.5810000         -0.091687974012
  2     1.5800000         -0.180010443274
  2     0.5640000         -0.255703363785
  2     0.0734500          1.078277723416
S   1
  3     0.0280500          1.000000000000
P   3
```

```
  4     1.5340000          0.038003981035
  4     0.2749000          0.232032118583
  4     0.0736200          0.834631408456
P   1
  5     0.0240300          1.000000000000
D   1
  6     0.1239000          1.000000000000

li      3.0         0.0000           0.0000           6.0000
S   8
7  1469.0000000        0.000765601232
  7   220.5000000          0.005888932715
  7    50.2600000          0.029655553732
  7    14.2400000          0.109123162563
  7     4.5810000          0.282641784375
  7     1.5800000          0.452887111102
  7     0.5640000          0.274630956861
  7     0.0734500          0.009745923779
S   8
8  1469.0000000       -0.000224991961
  8   220.5000000         -0.001730563167
  8    50.2600000         -0.008791560880
  8    14.2400000         -0.033152565467
  8     4.5810000         -0.091687974012
  8     1.5800000         -0.180010443274
  8     0.5640000         -0.255703363785
  8     0.0734500          1.078277723416
S   1
  9     0.0280500          1.000000000000
P   3
  10    1.5340000          0.038003981035
  10    0.2749000          0.232032118583
  10    0.0736200          0.834631408456
P   1
  11    0.0240300          1.000000000000
D   1
  12    0.1239000          1.000000000000
```

Basis-B

```
li      3.0         0.0000            0.0000          0.0000
S   8
1  1469.0000000         0.000765601232
  1   220.5000000           0.005888932715
  1    50.2600000           0.029655553732
  1    14.2400000           0.109123162563
  1     4.5810000           0.282641784375
  1     1.5800000           0.452887111102
  1     0.5640000           0.274630956861
  1     0.0734500           0.009745923779
```

```
S   8
2  1469.0000000         -0.000224991961
  2   220.5000000         -0.001730563167
  2    50.2600000         -0.008791560880
  2    14.2400000         -0.033152565467
  2     4.5810000         -0.091687974012
  2     1.5800000         -0.180010443274
  2     0.5640000         -0.255703363785
  2     0.0734500          1.078277723416
S   1
  3     0.0280500          1.000000000000
S   1
  4     0.0074000          1.000000000000
P   3
  5     1.5340000          0.038003981035
  5     0.2749000          0.232032118583
  5     0.0736200          0.834631408456
P   1
  6     0.0240300          1.000000000000
P   1
  7     0.0074000          1.000000000000
D   1
  8     0.1239000          1.000000000000

li       3.0         0.0000           0.0000           6.0000
S   8
9  1469.0000000          0.000765601232
  9   220.5000000          0.005888932715
  9    50.2600000          0.029655553732
  9    14.2400000          0.109123162563
  9     4.5810000          0.282641784375
  9     1.5800000          0.452887111102
  9     0.5640000          0.274630956861
  9     0.0734500          0.009745923779
S   8
10  1469.0000000         -0.000224991961
  10   220.5000000         -0.001730563167
  10    50.2600000         -0.008791560880
  10    14.2400000         -0.033152565467
  10     4.5810000         -0.091687974012
  10     1.5800000         -0.180010443274
  10     0.5640000         -0.255703363785
  10     0.0734500          1.078277723416
S   1
  11     0.0280500          1.000000000000
S   1
  12     0.0074000          1.000000000000
P   3
  13    1.5340000          0.038003981035
  13    0.2749000          0.232032118583
  13    0.0736200          0.834631408456
```

```
P   1
  14    0.0240300          1.000000000000
P   1
  15    0.0074000          1.000000000000
D   1
  16    0.1239000          1.000000000000
```

Basis-C

```
li      3.0        0.0000           0.0000          0.0000
S   9
1  5988.0000000         0.000133195806
  1   898.9000000          0.001026509035
  1   205.9000000          0.005279761591
  1    59.2400000          0.020959812281
  1    19.8700000          0.066437667674
  1     7.4060000          0.166019058768
  1     2.9300000          0.315501808091
  1     1.1890000          0.394102355987
  1     0.4798000          0.191151004356
S   9
2  5988.0000000        -0.000077656259
  2   898.9000000         -0.000595364655
  2   205.9000000         -0.003032292029
  2    59.2400000         -0.012299272305
  2    19.8700000         -0.038898390070
  2     7.4060000         -0.103900377014
  2     2.9300000         -0.206846691413
  2     1.1890000         -0.366970200153
  2     0.4798000         -0.414865622550
S   1
  3     0.0750900          1.000000000000
S   1
  4     0.0283200          1.000000000000
P   3
  5     3.2660000          0.035544688557
  5     0.6511000          0.195796454763
  5     0.1696000          0.863995412271
P   1
  6     0.0557800          1.000000000000
P   1
  7     0.0205000          1.000000000000
D   1
  8     0.1239000          1.000000000000

li      3.0        0.0000          0.0000          6.0000
S   9
  9   5988.0000000         0.000133195806
  9    898.9000000         0.001026509035
  9    205.9000000         0.005279761591
```

```
  9     59.2400000          0.020959812281
  9     19.8700000          0.066437667674
  9      7.4060000          0.166019058768
  9      2.9300000          0.315501808091
  9      1.1890000          0.394102355987
  9      0.4798000          0.191151004356
S   9
10  5988.0000000        -0.000077656259
  10   898.9000000        -0.000595364655
  10   205.9000000        -0.003032292029
  10    59.2400000        -0.012299272305
  10    19.8700000        -0.038898390070
  10     7.4060000        -0.103900377014
  10     2.9300000        -0.206846691413
  10     1.1890000        -0.366970200153
  10     0.4798000        -0.414865622550
S   1
  11     0.0750900         1.000000000000
S   1
  12     0.0283200         1.000000000000
P   3
  13     3.2660000         0.035544688557
  13     0.6511000         0.195796454763
  13     0.1696000         0.863995412271
P   1
  14     0.0557800         1.000000000000
P   1
  15     0.0205000         1.000000000000
D   1
  16    0.1239000          1.000000000000
```

Basis-D

```
li      3.0         0.0000            0.0000          0.0000
S   9
1  5988.0000000         0.000133195806
  1   898.9000000         0.001026509035
  1   205.9000000         0.005279761591
  1    59.2400000         0.020959812281
  1    19.8700000         0.066437667674
  1     7.4060000         0.166019058768
  1     2.9300000         0.315501808091
  1     1.1890000         0.394102355987
  1     0.4798000         0.191151004356
S   9
2  5988.0000000        -0.000077656259
  2   898.9000000        -0.000595364655
  2   205.9000000        -0.003032292029
  2    59.2400000        -0.012299272305
  2    19.8700000        -0.038898390070
```

```
  2     7.4060000          -0.103900377014
  2     2.9300000          -0.206846691413
  2     1.1890000          -0.366970200153
  2     0.4798000          -0.414865622550
S   1
  3     0.0750900           1.000000000000
S   1
  4     0.0283200           1.000000000000
S   1
  5     0.0074000           1.000000000000
P   3
  6     3.2660000           0.035544688557
  6     0.6511000           0.195796454763
  6     0.1696000           0.863995412271
P   1
  7     0.0557800           1.000000000000
P   1
  8     0.0205000           1.000000000000
P   1
  9     0.0074000           1.000000000000
D   1
  10    0.1239000           1.000000000000

li      3.0         0.0000           0.0000           6.0000
S   9
  11   5988.0000000          0.000133195806
  11    898.9000000          0.001026509035
  11    205.9000000          0.005279761591
  11     59.2400000          0.020959812281
  11     19.8700000          0.066437667674
  11      7.4060000          0.166019058768
  11      2.9300000          0.315501808091
  11      1.1890000          0.394102355987
  11      0.4798000          0.191151004356
S   9
12  5988.0000000         -0.000077656259
  12   898.9000000          -0.000595364655
  12   205.9000000          -0.003032292029
  12    59.2400000          -0.012299272305
  10    19.8700000          -0.038898390070
  10     7.4060000          -0.103900377014
  10     2.9300000          -0.206846691413
  12     1.1890000          -0.366970200153
  12     0.4798000          -0.414865622550
S   1
  13     0.0750900           1.000000000000
S   1
  14     0.0283200           1.000000000000
S   1
  15     0.0074000           1.000000000000
P   3
```

```
  16     3.2660000          0.035544688557
  16     0.6511000          0.195796454763
  16     0.1696000          0.863995412271
P   1
  17     0.0557800          1.000000000000
P   1
  18     0.0205000          1.000000000000
P   1
  19     0.0074000          1.000000000000
D   1
  20     0.1239000          1.000000000000
```
